# Perspectives of maternal and child health care providers on their relationship with fathers during the first 1000 days of life: a Q-study methodology

**DOI:** 10.1186/s12884-026-09204-z

**Published:** 2026-05-16

**Authors:** Femke Hilverda, Zita Mast, Sushma C. Munshi, Justine van de Beek, Hilmar H. Bijma, Violet Petit-Steeghs

**Affiliations:** 1 Department of Socio-Medical Sciences, Erasmus School of Health Policy and Management, Erasmus University Rotterdam, Rotterdam, The Netherlands; 2 Department of Obstetrics and Gynaecology, Division of Obstetrics and Fetal Medicine, Erasmus MC - Sophia Children’s Hospital, University Medical Centre Rotterdam, Rotterdam, The Netherlands; 3 Department of Health Care Governance, Erasmus School of Health Policy and Management, Erasmus University Rotterdam, Rotterdam, The Netherlands; 4 Department of Care Ethics, University of Humanistic Studies, Utrecht, The Netherlands

**Keywords:** Maternal and childcare providers, Perspectives, Relationship-centred care, Fatherhood, Family-centred care

## Abstract

**Background:**

Research shows that maternal and child health care providers can play an important role in supporting men in the process of becoming a father and caring for a child. The way this professional support takes shape depends on health care providers’ relationship with fathers. Yet, insights into how health care providers perceive the relationship are currently limited. Therefore, the aim of this study is to explore the perspectives of health care providers in maternal and childcare on their relationship with fathers.

**Methods:**

A Q-methodology design was used with 31 statements. The participants (*n* = 15) consisted of maternal and child health care providers who regularly are in contact with fathers during the first 1000 days of their child’s life, such as midwives and obstetricians. This target group was selected since previous research highlights the importance of father involvement starting at an early stage and that in that stage father involvement in the care process is often lacking. Perspectives were based on statement raking grouped through Principal Component Analysis (PCA) and interpreted using interview data.

**Results:**

Four perspectives on how health care providers perceive their relationship with fathers were found. These perspectives focus on: (1) the leading role of healthcare providers which lacks attention to fathers; (2) joint agreements and trust without paying specific attention to fathers; (3) equal interaction between healthcare providers, mothers and fathers; and (4) supporting fathers to take up their unique role.

**Conclusions:**

Our results show substantial differences between maternal and child health care providers in how they view their relationship with fathers, attributing varying roles to themselves and the fathers. Interventions in maternal and child healthcare aiming to increase paternal involvement should take these perspectives on the care relationship into account as not all maternal and child health care providers prioritize active involvement of (future) fathers. This implies that a (cultural) change in attitudes of care providers is needed to enable father involvement in maternal and child healthcare.

## Background

Maternal and child health care during the first 1000 days (from conception to child age 2 years) in the Netherlands has traditionally focused on the mother as the most important caregiver of the child(ren)^1^ [1–4]. Contextual and structural factors, such as reimbursement and legal context, are thus focused on the pregnant individual. It is, therefore, not surprising that the focus of health care providers is mostly directed at the (future) mother [5, 6] and most are not trained to involve fathers [7]. As a consequence, care providers spend little time specifically aimed at supporting fathers [6]. Because birth and childcare providers often forget to or are unsuccessful in involving fathers [8–10], (future) fathers often experience being disregarded by care providers or feel alienated during prenatal care consults [4, 8, 9, 11–16].

Yet, the importance of (future) fathers’ involvement in birth and child care has been broadly emphasized in both research and practice [11, 13, 17–20]. Berg points to the particular importance of involving fathers at an early stage (e.g. [21]. In line, research shows the benefits of the involvement of (future) fathers in maternal and child health care for both parents as well as children [15, 17, 22–25]. For parents, fathers’ involvement helps lower parental stress - particularly for mothers, since (future) fathers can play an assisting role during pregnancy, birth, and childcare [26] - and enhances relationship satisfaction [27–29]. For children, father involvement has been positively associated with emotional wellbeing, cognitive growth, and academic success [28–33]. Additionally, involved fathers generally experience positive outcomes, like better mental health [34–36].

Given the positive effects of involving (future) fathers during pregnancy and after birth, it is essential to encourage maternal and child healthcare providers to engage (future) fathers [32]. Father involvement can be facilitated by a good relationship between healthcare provider and father [37, 38]. Previous research shows that while most fathers view their role as secondary [39], (especially first-time) fathers perceive being actively engaged by care providers as valuable in assisting them to be supportive during the pregnancy, bond with their (unborn) child, and share worries or joy with their partner [26]. Active involvement of fathers is also expressed as a wish of (future) mothers [4, 12]. However, fathers often experience a lack of support from by professionals [40]. Moreover, oftentimes the support provided is not in line with fathers’ needs. Support is regularly perceived by fathers as unsatisfactory - for example, where fathers often prefer practical support, providers tend to focus on emotional support [8, 40, 41]. At the same time, research indicates that health care providers do not see it as their job to involve fathers [20] or experience educational and resource-related barriers in doing so [7].

It remains, however, largely unknown how healthcare providers perceive father involvement in maternal and child health care, how they view their relationship with the father of the child, and what aspects they perceive as important regarding this relationship. To fill this gap, the aim of the research was to explore perspectives of maternal and child health care providers regarding their relationship with fathers. In this way, strategies can be developed for increasing the involvement of fathers. This study explored the following research question: “*What are the perspectives of maternal and child health care providers on their relationship with fathers?*”

To answer this question, a Q-methodology was chosen [42, 43]. This methodology is particularly relevant to use for mapping individuals’ opinions and viewpoints regarding a certain topic, such as perspectives on care relationships [44]. Our study explores which perspectives maternal and child healthcare professionals hold regarding their relationship with (future) fathers.

## Methods

To explore the perspectives of maternal and child healthcare providers on their relationship with fathers, a Q-methodology was used. This mixed-method approach combines a quantitative ranking of statements with a qualitative interview to further explore personal experiences, values, and beliefs in relation to the ranking [45]. Q-methodology serves the purpose to systematically examine perspectives or viewpoints [43] and thus can perfectly be used to answer our research question. A Q-methodology study entails the following three steps: (a) design of the Q-set, (b) administering the Q-sort, and (c) statistical analysis and factor interpretation using both quantitative ranking scores and qualitative data from the interviews. The checklist for reporting a Q-methodology was used [42].

### Design of the Q-set

The statements, referred to as the Q-set, were derived from previous research on the relationship between healthcare providers, clients, and informal caregivers [46] and discussions among the research team. The initial Q-set, as used by van Muijden et al., contained 28 statements and was largely based on the relationship-centered ‘senses’ framework [47]. To make sure the Q-set fully covered all aspects of the care relationship, additional literature on father involvement in maternal and child healthcare was explored. In addition, the Q-set was discussed within the research team consisting of both healthcare professionals (gynecologist, nurse) as well as experts (researchers) in father involvement research. Thereafter, three statements were added to the initial set. One of these statements encompassed the relation between father and mother, the other two were about practical support and humor, aspects that were deemed important by fathers in their relationship with maternal and child healthcare providers. All statements were rephrased to fit the context of the current study keeping in mind that the relationship between care provider and father was central. The final Q-set (shown in Table 2) consisted of 31 statements. To enhance acceptability and comprehensibility, two test interviews were held. After two test interviews, one statement (statement 16 about making one’s own choices) was rephrased to better specify the father’s role (and his ability to make his own choices) in the statement. To maintain consistency in explaining the statements, a description was established in advance and provided as verbal explanation when requested.

### Participants

To capture the range of potential perspectives, a purposive sampling approach was used to select participants with well-formed opinions and substantial knowledge on the subject [48–50]. This refers both to the specific research focus of fathers’ relationships with healthcare providers as well as to the broader field of maternal and child health in which these relationships are situated. Although initially existing professional connections of the research team were used for participant recruitment, participation was voluntary and independent of prior relationships. Additionally, healthcare providers in the province South Holland were directly contacted via email or phone. Various types of maternal and childcare providers delivering care services during the first 1000 days of a child’s life, were approached. In the Netherlands, several types of healthcare providers are relevant during the first 1000 days of life [51–53]. Women who are considered to have a low risk of complications during the perinatal period generally receive care from a community midwife. Women who are considered to have an increased risk of complications during the perinatal period receive obstetrician-led care, provided by medical midwives, doctor or obstetricians. Additional caregivers, such as a pediatric nurse or pediatrician may be involved if more care is needed. During the postpartum period, parents receive assistance from maternity nurses for about one week at home, and regular check-ups from community midwives. After the postpartum period, preventive youth healthcare workers provide preventive childcare until the child turns 18. If a family needs extra support, specific interventions are available, such as home visits by a family nurse. 

The inclusion criteria focused on individuals with direct professional experience or expertise in the field of maternal and child health. Maternal and childcare providers were included in case they interacted professionally with (future) fathers during service delivery in the Netherlands. Characteristics such as gender, age, profession, and work experience were also considered in the selection process to obtain a diverse group. Furthermore, participants were recruited using the snowball method, specifically by asking participants if they knew other professionals with different viewpoints on the subject [48]. 

Participants were recruited until no new views on the topic emerged from interviews, indicating data saturation. The final sample for this study comprised of 15 participants. The demographic information of the participants is presented in Table 1. Since working in maternal and childcare is mostly a female profession, most participants identified as women, but we were still able to include two participants identifying as men. 

**Table 1:** Participant demographics (*n*=15)

Demographics	Frequency
Gender	
Male	2
Female	13
Age	
18-25 years	2
26-35 years	2
36-45 years	3
46-55 years	3
56-65 years	4
Over 65 years	1
Occupation	
Midwife*	2
Obstetricians	2
Preventive youth healthcare nurse	4
Preventive youth healthcare pediatrician	2
Maternity nurse	2
Other**	3
Years of relevant work experience	
0-2 years	0
2-4 years	4
5-10 years	2
10-15 years	0
More than 15 jaar	9

*One of the two midwives was no longer actively practicing as such but was currently working as a specialist and trainer in the field of father involvement

**Pediatric Nurse (*n* = 1) and Family Nurse (*n* = 2)

### Q sort procedure

Participants were contacted via email or phone to schedule an appointment. Depending on the participant's preference and practical feasibility, 8 interviews took place online, while 7 were conducted in person at a location most convenient for the participant. A script was used to ensure consistency. Prior to the interviews, participants were informed about the purpose and procedure of the research via email. They got the opportunity to ask questions via email and at the start of the interview before they agreed to participate. Participants provided informed consent online, agreeing to participate in the research and the usage of the data and audio recording. One participant declined audio recording, so extensive notes were taken and used for interpretation. After signing the online informed consent form, participants were asked to provide some background information, including gender, age, occupation, and years of (relevant) work experience, through an online questionnaire using Qualtrics. Participants were informed that they could withdraw at any moment, regardless of prior consent, without reason or consequences. 

**Fig. 1:**
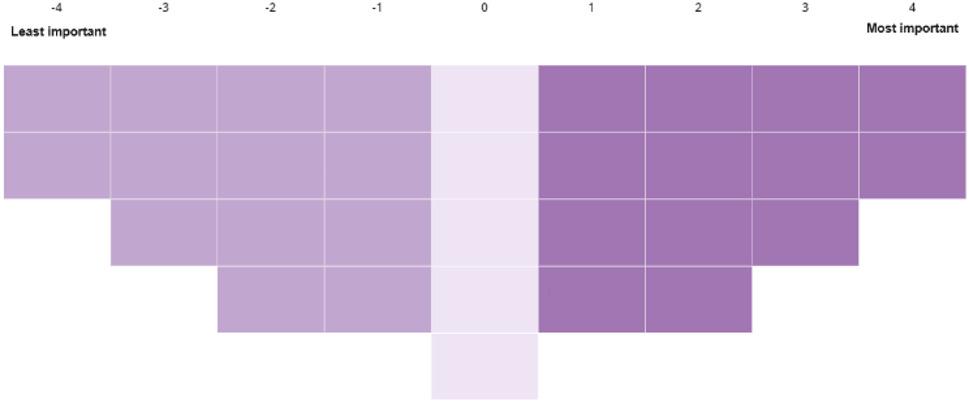
Q-grid

Subsequently, participants were presented with the Q-sort. They sorted the statements using an online tool (Q Method Software). Initially, participants were asked to pre-sort the statements into three categories: ‘not or least important’, ‘neutral’, and ‘very or most important’. Participants were encouraged to read the statements out loud and clarify their placement choices. Following the pre-sort, participants positioned the statements on a standardized Q-grid ranging from – 4 (least important) to + 4 (most important) (see Fig. 1). They were instructed to place the statements they found ‘very or most important’ on the right side of the grid, while the ‘not or least important’ statements were positioned similarly on the left side. This process continued until all statements were sorted, and the grid was filled. Throughout and after the Q-sort, participants were asked questions to provide explanations for their selections. The entire procedure lasted approximately 45-60 minutes per interview. The interviews were conducted in April 2023.

### Data analysis

Quantitative data were analyzed using Q Method Software. Participants’ rankings of statements were compared and grouped through Principal Component Analysis (PCA) [48, 49]. The PCA aimed to distinguish perspectives by grouping participants with similar views together, while distinguishing between participants with dissimilar responses. The number of perspectives was determined based on the Scree plot (the ‘elbow’ of the curve indicates the number of factors) and the Kaiser-Guttman criterion (the number of Eigenvalue scores that exceeds the value of 1 is equal to the number of factors). Additionally, preferably at least three participants should significantly load on each perspective (*p* < 0.05) and the perspectives with corresponding participants should make sense based on the qualitative interview data. After determining the number of perspectives, factor rotation (varimax) was used to maximize variance between the components, allowing participants to be optimally associated with one perspective [54]. The interpretation of the perspectives was based on distinctive, characteristic and consensus statements that were found with the quantitative analysis, along with the qualitative data obtained from interviews. Interviews were transcribed verbatim and field notes were made during the interviews and sorting process. In addition, the results were discussed among the research team to validate the perspectives.

## Results

The overall results of the PCA point to four distinctive perspectives. However, the fifth Eigenvalue also slightly exceeded 1 (1.04), but considering the Scree plot, explained variance, and participant loadings a four factor solution was preferred. These four perspectives account for 66% of the total explained variance in ranking, which is considered considerably high within Q-methodology [43, 55]. This indicates that the factor solution provides a robust representation of the range of viewpoints expressed by participants. The rest variance (34%) is a normal part of Q-studies and represents the personal and unique variation between respondents. Fourteen of the fifteen participants had significant loadings on a perspective (*p* < 0.05). As one Q-sort did not load significantly on any factor and therefore could not be assigned to a shared viewpoint, interview data of this participants was not used when interpreting the perspectives.

Table 2 shows the statement ratings per perspective. These ratings are based on the combined rankings of all participants who load on a perspective. In this way, weighted averages were used to create how a typical participant with a certain perspective would rank the statements [43]. In addition, this table shows the distinctive statements per perspective and consensus statements among all perspectives. Respondents who hold one of the first two perspectives contribute no explicit role for the father during service delivery, while the other two perspectives envision explicit inclusion of (future) fathers. The first perspective puts the healthcare provider in charge, giving no specific attention to father; the care relationship is focused on the care for mother and child (perspective 1). Healthcare providers with the second perspective focus on the process rather than having a clear view on the leading actors in the care relationship. Specifically, little attention is paid to the father. Their focus is on rules, trust, and agreements between actors (perspective 2). In the third perspective, the need for shared decision making and the importance of equally involving the father and mother is emphasized (perspective 3), while the fourth perspective emphasizes the inclusion of the father to empower him in his unique role (perspective 4). Each perspective is described in detail below.

**Table 2:** Q-sort values per perspective

#	Statements	Perspective 1	Perspective 2	Perspective 3	Perspective 4
1	Attention for the father’s knowledge	-1	-2	2*	-1
2	Attention for the life story of the father	-4	-2	3*	-2
3	Attention to the care that the father previously received	0	-3*	0	3*
4	Attention for the relationship between mother and father	-3*	2	3	-1*
5	Having mutual understanding	1	-1	1	-2
6**	Being approachable for each other	-1	-2	0	-1
7	The father’s satisfaction with his own contribution	-1	-3*	0	3*
8	The healthcare provider being there for the father	1	-1*	1	4*
9	That the relationship stays businesslike	0*	-4	-4	-4
10	Allowing both father and healthcare provider to contribute to the care for mother/child	-1	0	-3	0
11	The healthcare provider’s feeling of being capable of providing good care	3*	-2	-3	1*
12	Everyone adhering to agreements	0*	4*	-1	-3
13	Allowing deviation from the rules	0	0	-2	-2
14	Clarity on organizational rules	-3	2*	-3	-4
15	Clarity on division of responsibilities	4	3	-2	0
16	The father being able to make individual choices	-3*	-1*	2	2
17	Discussing vulnerabilities and feelings	1	1	4*	1
18**	Trusting one another	4	4	2	2
19**	Appreciation one another and giving compliments to each other	-2	-1	-1	-2
20**	Being able to set boundaries	0	1	0	0
21	Attention to the father’s role during pregnancy, birth, and parenting	-4*	0*	4	4
22**	Being open and honest with each other	3	2	2	2
23	Consulting on what the appropriate care is	1	1	3*	0
24	The healthcare provider in the driver seat	3*	-4	-2*	-3
25	Being considerate of each other	2	1	-1	0
26	Willingness to learn from each other	-2	0	-2	-3
27	Trusting the professional’s knowledge	2	3	1	1
28	Discussing expectations with each other	2	3	1	1
29	Striving for the same goal	2	0	0	2
30	Providing practical support	-2*	2	-4*	3
31	Space for humor	-2	-3	-1	-1

*Distinctive statement

**Consensus statement

### Healthcare provider has leading role; father is not important (perspective 1)

Following this perspective, the healthcare provider is in charge (#24; +3) in the care relationship. One participant (obstetrician 1) explains: “What is most important for me is that it’s clear who is responsible for what and that the healthcare provider takes the lead. I think that during labor, it’s clear that I am in charge.” Even though clarity about responsibilities (#15; +4), such as the leading role of the healthcare provider, is found important transparent communication about the process of care-provision is according to participants not always possible or necessary. More value is placed – compared to the other perspectives - on keeping the contact businesslike (#9; 0). To be able to be in charge, healthcare providers with this perspective believe that it is also important that the healthcare provider feels that they can provide good care (#11; +3). This requires mutual trust towards each other, which is valued highly in the ranking (#18; +4).

Moreover, healthcare providers with this perspective believe that they should mainly support the mother and/or child, and less the father. During the interviews they explain that they feel the mother and/or child are their priority since they think fathers have no clear role in medical birth or childcare. This is also why they attach less importance to the father’s life story (#2; -4) in the ranking. Healthcare providers explain in the interviews that only if there is a potential problem, such as a history of mental health issues, is it good to pay attention to the life story of the father as a healthcare provider. This also applies to the relationship between father and mother (#4; -3); participants say that this is only important if something relevant is at stake. Furthermore, the father’s role during pregnancy, birth, and parenting (#21; -4) and his ability to make own decisions (#16; -3) are ranked as unimportant. One participant (obstetrician 2) explains: “The role of the father doesn’t matter to me; it’s between them [parents]. I can have an opinion, but it doesn’t matter. If it’s not a threat to the child, I don’t care.” and further explains: “That the father can make his own choices is not always relevant. It’s about the mother and child.” From this perspective, the decisions of mother and caregiver are viewed as more important than preferences of father. One participant (pediatric nurse 1), for example, states: “I think the father has something to say, but he doesn’t always have a say in everything.”

To sum up, in this view, the healthcare provider leads the process with a professional approach, emphasizing their ability to provide good care for mother and child. Healthcare providers with this perspective attribute a less important role to the father; giving little attention to the father’s role, story, or the relationship between the parents. Three participants loaded on this perspective, including two obstetricians and one pediatric nurse, explaining 18% of the total variance.

### Focus on joint agreements and trust, lack of specific attention for fathers (perspective 2)

In this perspective, much attention is given to expectation management in the care relationship. That implies that everyone adheres to agreements (#12; +4) and that is discussed who is responsible for what (#15; +3). During the interviews, participants explain that discussing expectations’ (#28; +3), but also ‘transparency of the rules of the healthcare organization’ (#14; +2) can help speaking up if someone does not meet agreements and therefore reinforce responsibilities, trust (#18; +4), and specifically trust in the healthcare provider’s knowledge (#27; +3), is also very important in this perspective. One respondent (maternity nurse 1) indicates that trust is “the foundation of everything”. Another participant (youth healthcare nurse 1) further explains: “If a father does not trust you, you cannot provide proper care, even though you may do it well”.

In contrast to perspective 1, it is – in this perspective - not important that the healthcare provider takes the lead. One participant (youth healthcare nurse 2) says: “I don’t necessarily need to take the lead in a specific case. I find it more important that we make it work”, implying that the collaboration process is important and that both the father and healthcare provider can contribute. The ranking also shows that it is not important that the contact remains businesslike (#9; -4), in contrast to the first perspective. A participant (maternity nurse 2) shares: “The birth of a child, there is nothing business-like about it.”

Even though healthcare providers with this perspective say that the father is sometimes forgotten, or as one participant (maternity nurse 1) states “he actually stands on the sidelines”, these participants prefer general statements about aspects of a good relation over statements that are specifically about their relationship with fathers in the ranking. A participant (maternity nurse 2) explains: “A father certainly participates, of course, but that is not the emphasis.” Consequently, little importance in the ranking is given to the statements ‘the father being satisfied with his own contribution’ (#7; -3), ‘attention to the father’s experiences with previously received care’ (#3; -3), ‘that the healthcare provider is there for the father’ (#8; -1) or ‘that the father can make choices’ (#16; -1).

To sum up, in this perspective little attention is paid to the relationship between care provider and father, specifically. The perspective distinguishes itself by emphasizing the importance of managing expectations and making agreements, with trust being a crucial aspect within this process. Participants express that these characteristics are fundamental prerequisites for a good care relationship and are therefore deemed most important. A good care relationship in turn leads to good outcomes. It remains unclear who should take the lead in the care process. Four participants loaded on this perspective, including two maternity nurses and two youth healthcare nurses, and it explains 8% of the total variance.

### Equal interaction between healthcare providers, mothers and fathers (perspective 3)

In this perspective, attention in the care relationship is paid to both mothers and fathers. Healthcare providers who load onto this perspective believe that both parents (and their relationship: #4; +3) are fundamental to child development. Fathers’ role during pregnancy, birth, and parenting (#21; +4) is ranked highly and perceived as just as important as the role of the mother. One healthcare provider with this perspective (family nurse 1) mainly speaks about “parent” and rarely uses the term “father”. Emphasis is placed on supporting parents, and this also means involving the father. Healthcare providers with this perspective believe that fathers and mothers must be equally involved by healthcare providers. To involve fathers, providers feel that it is important to pay attention to their life story (#2; +3), such as the experiences of mental health issues, and the knowledge of the father (#1; +2). One healthcare provider (family nurse 1) says: “Attention to the father’s life story is something I find very important… the background of parents naturally influences how they handle their own baby and how they perceive certain aspects.”

This perspective further distinguishes itself by the importance placed on the personal approach in the care relationship shown by the ranking of the statements on ‘discussing the appropriate care’ (#23; +3) and ‘discussing vulnerabilities and feelings’ (#17; +4). Vulnerabilities and feelings can be expressed by both the father and the healthcare provider, as one participant (youth healthcare physician 1) explains: “Why shouldn’t a healthcare provider be allowed to say that, if it’s something that affects them… I think that only improves your connection with a client, a parent, or whoever. Because you also show them, hey, you’re just a human, you’re not untouchable.” While healthcare providers with this perspective express that ‘you’re just human’, they do feel it is important professional distance should be kept. However, contact does not have to be businesslike (#9; -4), a light-hearted attitude can assist in establishing a connection.

In contrast to the perspective 1 and like perspective 2, it is not that important that the healthcare provider takes the lead (#24; -2). One healthcare provider (midwife 1) explains: “In acute situations, of course, but otherwise, I prefer it when parents themselves say what they want during childbirth, and that they ask me when they think that it’s necessary.” In the ranking, less importance is also placed on providing practical support (#23; -4). During the interviews, participants oftentimes link this to their job; it is not always their task to provide practical support.

To sum up, this perspective emphasizes the equal roles in the care relationship. This also implies involving the father by understanding the father, his story, knowledge, and role, as well as the relationship between the father and mother. Emphasis is placed on the needs of both parents. Decisions about care are made through discussions where the healthcare provider does not take charge. This view also values the healthcare provider’s role, recognizing their vulnerabilities and emotions. Four participants significantly loaded on this perspective, including two family nurses, a midwife, and a youth healthcare physician, explaining 30% of the total variance.

### Healthcare provider supports fathers to take up their unique role (perspective 4)

This perspective stands out by focusing on the father and his unique role during the pregnancy, birth and in childcare. Healthcare providers with this perspective not only believe that the father and mother are equally important, like perspective 3, but also highlight the uniqueness of the role of the father, like one respondent (midwife 2) indicates: “The father is not the partner, but one of the two parents with his own role.” Characteristic for this perspective is the importance placed on this role in the ranking (#21 + 4). The relationship between father and mother is not that important at an individual level in the ranking (#4; -1), but according to the interviews with the healthcare providers much more on a broader level beyond the consulting room.

Healthcare providers with this perspective explained that times are changing. That is, they describe that fathers take up their role as caregivers for their children and are more regularly seen attending appointments alone. Providers with this perspective argue that a change towards more inclusive care in which fathers are closely involved in childcare and their needs are considered, is needed. They feel that the societal views on parenting influence how healthcare providers interact with parents, and that traditional views lead to overburdened mothers and as well as fathers who are not empowered.

Healthcare providers with this perspective express that much information and guidelines are, however, still focused on mothers. Consequently, healthcare providers focus too much on the mother, while they should also be available for the father (#8; +4) in a serving role, not a leading role (#24; -4). A participant (midwife 2) stated: “I’ve noticed that in the entire guidelines and basically everything we do as healthcare providers around pregnancy, birth, and upbringing, we still collectively set a tone that the mother is the primary caregiver, and thereby, we pay too little attention to the father during that period.” During the interviews it became apparent that these protocols and rules are less important to participants with this perspective, they feel that it is most important that both parents have major input.

This perspective places fathers’ needs and his perspective central in the ranking by paying attention to the father’s experiences with previously received care (#3; +3) and by aiming that the father is satisfied with his own contribution and choices (#7; +3). One participant (midwife 2) explains: “I believe that if healthcare providers pay more attention to past experiences of fathers, as we do with mothers, we can quickly learn and adapt to the father’s wishes, needs, and expectations. Because then, we quickly hear what we could have done differently last time, and we can try to adjust immediately.” This participant also underlined the importance of practical support for the father (#30; +3) as “men tend to have a practical mindset, focused on taking action”. Following the respondents who hold this perspective’s ranking, fathers should be allowed to make their own choices (#16; 2) since, as addressed during the interviews, father’s choices might differ from the choices of mothers.

To sum up, participants that were strongly associated with this perspective express a wish to include the unique role of the father in the care relationship. While this perspective shares some similarities with perspective 3 by acknowledging the importance of the father, the key difference lies in the healthcare provider’s perceived own role, which is more serving, and in perceiving (a need for) a broader societal change. Here, the emphasis is on the father, his role in the family, the healthcare provider’s support for him, and his satisfaction with his involvement. The healthcare provider aims to align with the father’s wants and needs. Three participants loaded on this perspective, including a youth healthcare nurses, a midwife and a youth healthcare physician, and it explains 10% of the total variance.

### Consensus statements

In addition to distinctive and characteristic statements, consensus statements were identified. Consensus statements are similarly viewed across perspectives. For example, the statement ‘being able to set boundaries’ (#20) is considered ‘neutral’ within all perspectives. Additionally, all perspectives emphasize the statements ‘that trusting each other’ (#18) and ‘being open and honest with each other’ (#22) are crucial aspects.

Furthermore, all healthcare providers agree that it is less important to be available to each other (#6) and to show appreciation and give compliments to each other (#19), mainly because this is part of their job and not expected in return of fathers or mothers.

## Discussion

This study examined the perspectives of maternal and child health care providers on their relationship with (future) fathers in the context of healthcare providers’ care provision. To do so, a Q-methodology design was carried out with care providers who are in contact with fathers during the first 1000 days of life of their child. Our results show four distinctive perspectives. In these perspectives, the care relationship was characterized by (1) the leading role of healthcare providers in which little emphasis is paid to the father; (2) joint agreements and trust where little attention is given to the father specifically; (3) the equal interaction between healthcare providers, mothers and fathers; and (4) the support for fathers to take up their unique role.

Within these four perspectives, the role of both the care provider (level of taking the lead) and the father (amount of attention paid to him) differed. In line with earlier research, showing that healthcare providers do not always perceive it as their task to involve fathers [7, 20], we found two perspectives with no specific role attributed to the father. That is, in the first perspective, the healthcare provider is in charge and is focused on the care for mother and child, while there is little or no attention for the father. Here, the healthcare provider has a clear goal, aims to provide quality of care for mother and child in the short term, and wants to feel content with their own contributions.

Similarly, in the second perspective, the focus is not necessarily on the father. Healthcare providers with this perspective, lack a clear view about the leading actors and rather focus on rules and agreements between actors. The emphasis is on clarity and establishing a solid foundation of agreements and expectations upon which the rest of the healthcare relationship can be built. A good process likely leads to good outcomes. However, in cases of absent contact or parental conflict, these providers are likely to prioritize the mother’s needs, as she is their official client and thereby the primary point of agreement [7]. In these cases, healthcare providers may not focus on the father out of fear it may damage their relationship with the client, by not aligning with her needs or imposing on her autonomy [40].

We extend insights from previous literature by two perspectives that focus explicitly on the role of the father. The third perspective highlights the need for shared decision making and the importance of involving both father and mother equally. This is deemed important because involvement of both parents is associated with better child development (in the long term), which is also highlighted in previous literature [28, 30–32]. The fourth perspective emphasizes the inclusion of the father to empower him in his unique role. In this perspective the healthcare provider takes a serving role to support the father.

Interestingly, the perceived relationship seems to differ depending on the profession of the provider and stage in which the healthcare providers encounter fathers. That is, the first perspective was shared by participants who all worked in the hospital, which might imply that this perspective is mostly prevalent in medical settings. In line with this, previous research showed that women perceive secondary/tertiary hospital care to be less responsive to client needs compared to community based primary care [4]. This could mean that in medical settings, in which there is a high(er) risk of complications, healthcare providers are more used to taking the lead and focus less on specific wishes of the clients, such as actively involving fathers, since urgency takes over [4].

In contrast, care providers with the third or fourth perspective seem to focus more on the social setting of clients and oftentimes have occupations that are more directly in contact with other actors than the mother, for example the maternity or family nurse who makes home visits and meets the father, other children, family members or friends. The difference in focus on the care situation of the mother and child versus the focus on the family including the father, seems to overlap with the difference between the medical and social model. While the medical model focusses on individual pathology, the social model is a holistic approach, emphasizing the importance of the social aspects of health such as the context of the mother and the family, and not merely the physical aspects of illness and health [56]. Also, the focus is not necessarily on the health outcomes of the mother, but rather on the satisfaction of both mother and family with the care process and overall well-being [57–59].

The differences in perspectives regarding the role of both care provider and father, the focus on process or outcomes, and the nature of contact (formal or not), are important to acknowledge when developing policy strategies to stimulate father involvement. However, while some maternal and child healthcare providers give less priority to including fathers, it does not necessarily mean that these care providers do not realize the importance of an involved father. It may well be that they simply do not have sufficient time to involve them or do not consider it their responsibility [20]. Moreover, healthcare providers might not feel the space to actively involve fathers given the Dutch healthcare regulations in which the (future) mother is described as the client, while the caregiver does not have a formal relationship with the father [2, 3]. This means that in the Dutch context, a change in protocols, regulations, finance, and procedures might be needed to stimulate father involvement in maternal and child healthcare settings since healthcare providers officially do not have time and resources to engage with fathers.

Our findings highlight the variation in perspectives on the importance of father involvement and hereby extend the results of recent studies [7, 20, 60]. While these studies also shed light on provider’s attitudes on father involvement, they do so on providers operating in specific and different phases: during pregnancy or in early childhood. The current study illuminates findings from a variety of healthcare providers who support parents throughout pregnancy, child birth and early childhood. As a consequence, it shows more variation among perspectives, likely linked to dominant models on health within professions. Furthermore, we had a novel approach on focusing on the relationship healthcare providers believe to have with fathers. Thereby, we show that beyond attitudes on father involvement or contextual factors impeding or stimulating it, father involvement by healthcare providers is also influenced by provider role conceptions.

### Strengths, limitations, and future research

This study provides insights into the perspectives and experiences of healthcare providers in relation to working with fathers, a perspective that is oftentimes overlooked. Some limitations are, however, worth mentioning. Firstly, subjectivity in both the selection and interpretation of perspectives might have occurred. That is, looking for example at the Eigenvalues a five factor solution might have been preferred, while other indicators (such as Scree plot, qualitative data) made the authors select the four factor solution. The first and second author worked in close collaboration on the analyses of both the ranking and interviews, and discussed the findings within the whole research team, which led them to conclude four perspectives resembled the data best. Also, social desirability in the found perspectives seems unlikely since some participants perceived father involvement less important. In addition, the statements of the Q-set have been used in previous research and further developed by cross-referencing existing literature and through direct input from healthcare providers and test-interviews, ensuring validity and reliability of the findings.

Another potential limitation of this study is that the research sample is limited, and the data collection period was relatively short. However, there are multiple reasons we believe that this is not really problematic in our specific case. Most importantly, in Q-methodology studies only a small number of participants is needed to explore a variety of perspectives on a certain topic, considering participants are purposively selected and represent a range of different opinions [43]. In our study, we were able to detect a variety of perspectives and include a diverse range of healthcare providers. Comparison with the literature and consultation with professionals working in birth and childcare did not provide us with any indications of missing perspectives. The result of also finding perspectives that pay less or no attention to fathers, strengthen this belief. Moreover, data saturation occurred since no new information was shared in the final interview implying our sample was sufficient to detect four distinctive perspectives among the research participants. The number of four perspectives is also in line with expectations based on the number of statements and participants, as proposed by Webler’s framework [50], which further indicates the high likelihood of not missing any perspective.

Furthermore, we only focused on health care providers in maternal and childcare, excluding the perspectives of fathers themselves. While some studies on fathers’ perceptions on father involvement in prenatal care exist (e.g. Walsh et al., [26]), a follow-up study using the same Q-set focusing on the perspectives of fathers explicitly about their relationship with maternal and child healthcare providers could provide valuable insights into optimizing care delivery. Additionally, further research specifically examining the alignment between these perspectives, as well as exploring the viewpoints of mothers, would be recommendable. Besides, this study presents an interesting starting point to further explore differences in the perspectives across certain occupations. For example, it was observed that all participants who loaded on perspective 1 worked in the hospital, which might imply that this perspective is mostly prevalent in medical settings. Based on Q-methodology, it is, however, not possible to draw conclusions about this since its goal is not to generalize. Therefore, a Q2S study, a survey based on results of a Q-study in which perspectives are used to examine representativeness of them in a large sample, could be performed using the perspectives of our study presenting them to a (large) representative group and examine possible relationships between the perspectives and characteristics of care providers.

## Conclusions

While maternal and child healthcare providers involved in the first 1000 days of life may play an important role in stimulating father involvement, we found that there is a variety of perspectives of healthcare professionals on their role in involving fathers. Not all care providers perceive their relationship with fathers as a priority. Although some healthcare providers envision an equally important role for fathers as mothers in relation to care providers, or even want to serve fathers to empower them in their unique role, some healthcare providers focus on taking the lead in providing care for the mother and child or mainly focus on rules and procedures. Interventions in maternal and child healthcare aiming to increase father involvement need to take these differences in perspectives on the care relationship into account. Not all healthcare providers give father involvement priority, implying that a (socio-cultural) change in attitudes of care providers is needed to enable father involvement in maternal and child healthcare. Moreover, even if healthcare providers want to involve fathers, current protocols, reimbursement, and procedures might be a barrier for actively engaging fathers.

## Data Availability

The quantitative (ranking scores) and qualitative (interview transcripts in Dutch) data of the current study are not publicly available due to privacy concerns but are available from the corresponding author on reasonable request.

## Footnotes

1 In this paper we speak about ‘mothers’ meaning all persons who (are going to) give birth to a (future) child. When we speak about ‘fathers’ we mean everyone who identifies as a father in the life of a (future) child, including non-biological fathers or foster parents.
